# Age at Initial Surgery and Surgical Burden in Congenital Spinal Deformity

**DOI:** 10.3390/medicina62061053

**Published:** 2026-05-28

**Authors:** Seidali Abdaliyev, Daniyar Yestay, Olzhas Bekarissov, Sergey Vissarionov, Dina Saginova, Murat Baidarbekov, Serik Serikov

**Affiliations:** 1 National Scientific Center of Traumatology and Orthopedics Named After Academician N.D. Batpenov, 010000 Astana, Kazakhstan; 2 The Institute of Life Sciences, Karaganda Medical University, 100000 Karaganda, Kazakhstan; 3 National Medical Research Center for Pediatric Traumatology and Orthopedics After G.I. Turner, Ministry of Health of the Russian Federation, 196603 Saint Petersburg, Russia

**Keywords:** congenital spinal deformity, surgical burden, pediatric spine surgery, multiple vertebral anomalies

## Abstract

*Background*: Congenital spinal deformities associated with multiple vertebral anomalies often require surgical correction during growth; however, the relationship between age at initial surgery and cumulative treatment burden remains insufficiently characterized. *Objective*: To evaluate whether age at first surgery is associated with surgical burden and radiographic outcomes in children with congenital spinal deformity treated with conventional posterior instrumented fusion. *Methods*: In this retrospective single-center cohort study, 32 children treated between 2019 and 2024 were stratified by age at initial surgery into two groups: ≤6 years (n = 13) and 7–12 years (n = 19). Planned staged procedures and growth-friendly techniques were excluded. Surgical burden was assessed as the total number of procedures, procedures per patient-year, and high surgical burden, defined as ≥3 procedures. Radiographic outcomes included postoperative Cobb angle and correction percentage. Adjusted analyses were performed using Poisson regression with log follow-up as an offset term, logistic regression, and linear regression. *Results*: Baseline deformity severity was similar between groups (mean preoperative Cobb angle, 45.2 ± 19.0° vs. 43.1 ± 21.6°; *p* = 0.61). Both groups showed significant within-group improvement after surgery (*p* < 0.001), with no significant between-group difference in correction percentage (61.5 ± 35.2% vs. 64.8 ± 30.6%; *p* = 0.78). The total number of procedures and procedures per patient-year were also comparable between groups (*p* = 0.21 and *p* = 0.58, respectively). However, high surgical burden was more frequent in the younger group (38.5% vs. 10.5%; *p* = 0.048). In adjusted analysis, older age at first surgery was associated with lower odds of high surgical burden (OR = 0.78; 95% CI: 0.61–0.99; *p* = 0.042), whereas no variable independently predicted correction percentage. *Conclusions*: Younger age at initial surgery was associated with a greater likelihood of high surgical burden, whereas the time-adjusted operation rate and early coronal correction were similar between groups.

## 1. Introduction

Congenital spinal deformities arise from failures of vertebral formation and/or segmentation and represent a heterogeneous group of conditions that may manifest as scoliosis, kyphosis, or kyphoscoliosis [1,2,3]. Their natural history varies according to deformity type, anatomical location, growth potential, and the presence of associated anomalies. Unlike adolescent idiopathic scoliosis, congenital deformities are often rigid, may progress early during growth, and frequently require surgical treatment to prevent worsening of coronal and sagittal imbalance. In addition, affected children commonly present with extracolumnar and intraspinal anomalies, further complicating treatment planning and perioperative management [2,4,5,6].

The timing of surgery in congenital spinal deformity remains one of the most challenging aspects of clinical decision-making [7,8]. Early intervention may limit deformity progression and reduce the development of secondary structural compensation; however, surgery in the immature spine may also initiate a prolonged treatment course associated with repeated interventions and greater cumulative burden for the child and family. Conversely, delaying surgery may reduce the number of procedures in selected patients, but this strategy carries the risk of further deformity progression and increasing technical complexity. Accordingly, both the timing of surgery and the choice of operative strategy remain heterogeneous, and no universal consensus exists regarding the optimal age for intervention across the full spectrum of congenital spinal deformity [7,8].

Recent literature has increasingly emphasized that success in pediatric spinal deformity surgery should not be assessed solely by the magnitude of radiographic correction. Reoperation risk, repeated hospitalizations, and overall treatment burden have emerged as clinically meaningful outcomes, particularly in growth-dependent spinal disorders. In congenital scoliosis, previous studies have shown that preoperative deformity severity, compensatory curves, and construct-related factors may influence the likelihood of additional surgery after limited fusion strategies. However, cumulative surgical burden has been less frequently examined from the perspective of age at initial surgery, particularly in clinically heterogeneous cohorts with multiple vertebral anomalies, in whom repeated procedures may be more common than in more morphologically uniform subgroups [5,9,10,11,12].

Another limitation of the existing literature is that many studies in congenital scoliosis focus on specific surgical techniques or narrowly defined morphological subtypes, such as hemivertebra resection cohorts, rather than on broader patient populations encountered in routine clinical practice. As a result, the available evidence does not fully address a clinically relevant question for both surgeons and families: whether earlier surgery meaningfully alters radiographic outcomes, or whether its principal consequence is a greater cumulative procedural burden over time [13,14,15].

The clinical novelty of the present study lies in its evaluation of cumulative treatment burden in a real-world cohort of children with multiple vertebral anomalies treated with conventional posterior instrumented fusion, while excluding planned staged procedures and growth-friendly strategies that could otherwise dominate the burden profile.

Therefore, the aim of the present study was to evaluate the association between age at initial surgery and cumulative surgical burden in children with congenital spinal deformities associated with multiple vertebral anomalies, while also comparing radiographic outcomes between children treated at ≤6 years and those treated at 7–12 years of age. We further sought to identify baseline factors associated with the cumulative number of surgeries and high surgical burden using multivariable regression models. We hypothesized that younger age at initial surgery would be associated with greater cumulative treatment burden, whereas the magnitude of radiographic correction would be broadly comparable across age groups.

## 2. Materials and Methods

### 2.1. Study Design and Setting

This retrospective single-center cohort study was conducted at the National Scientific Center of Traumatology and Orthopedics named after Academician N.D. Batpenov, Astana, Kazakhstan. The study included children with congenital spinal deformities associated with multiple vertebral anomalies who underwent surgical treatment between 2019 and 2024. Clinical, radiographic, and operative data were obtained from an institutional registry and supplemented by review of medical records when necessary. Before analysis, the dataset was reviewed for completeness and consistency, and non-patient entries or duplicated administrative rows were removed. After data cleaning, 32 complete patient-level records were available for final analysis.

### 2.2. Participants

Children were eligible for inclusion if they had a diagnosis of congenital spinal deformity associated with multiple vertebral anomalies, underwent initial surgical treatment during childhood, and had available preoperative and postoperative radiographic data. Patients were stratified according to age at initial surgery into two groups: ≤6 years and 7–12 years.

To preserve conceptual comparability of treatment burden, patients managed with planned staged procedures were not included. Likewise, growth-friendly techniques were not used in this cohort. Exclusion criteria included severe somatic comorbidities that could substantially affect the perioperative course or interfere with the interpretation of treatment outcomes. Associated congenital anomalies of other organs and systems were not considered exclusion criteria, as such conditions are common in this patient population. These included, among others, situs abnormalities, diastematomyelia, and minor congenital cardiac variants.

### 2.3. Surgical Strategy

To reduce treatment heterogeneity, the cohort was limited to patients treated with conventional posterior corrective surgery using instrumented fusion. The predominant operative strategy consisted of short-segment posterior spinal fusion with transpedicular instrumentation. In a limited number of cases, hooks were additionally used, mainly in the upper thoracic spine or at the cervicothoracic junction when required by local anatomical conditions. Growth-friendly constructs were not applied in the present series.

Information on repeat operations during follow-up was collected from operative records. Revision procedures, when they occurred, were assessed as part of cumulative surgical burden and were reviewed with attention to their clinical indication, including implant- or construct-related mechanical instability. In this cohort, reoperations related to recorded complications were mechanical in nature, including loosening and/or implant breakage.

### 2.4. Radiographic Follow-Up and Assessment

Radiographic follow-up was performed according to institutional routine practice. Standard postoperative radiographs were obtained for all patients at approximately 5–6 months after correction, repeated at 1 year, and subsequently performed during longer-term follow-up according to clinical need and routine surveillance. Additional imaging studies were obtained when there was clinical or radiographic suspicion of deformity progression or implant-related complications.

Coronal deformity was assessed using the scoliosis Cobb angle measured on preoperative and postoperative radiographs. For comparative analysis, the principal postoperative radiographic assessment was based on the standardized early follow-up examination at approximately 5–6 months after surgery. This time point was used as the primary reference for between-group comparison because it was available for all included patients and reduced bias related to unequal longer-term follow-up duration. Absolute correction was defined as the difference between the preoperative and postoperative Cobb angles. Relative correction was expressed as a correction percentage.

Kyphosis was evaluated both as a binary clinical-radiographic characteristic (present/absent) and, when paired measurements were available, as a continuous variable using preoperative and postoperative kyphosis angles.

### 2.5. Variables and Outcomes

The primary explanatory variable was age at initial surgery, analyzed both as a continuous variable and as a categorical variable (≤6 years vs. 7–12 years). Additional baseline variables included deformity localization, deformity type, preoperative Cobb angle, kyphosis status, and presence of associated anomalies.

The primary outcome of treatment burden was the cumulative number of surgical procedures per patient over the available follow-up interval. A secondary burden outcome was defined a priori as high surgical burden, corresponding to three or more surgical procedures. Very high burden (≥4 procedures) was also summarized descriptively. The number of hospitalizations was analyzed as an additional descriptive indicator of cumulative treatment burden.

Recorded complications during follow-up were additionally reviewed and classified according to documented complication type. Because only a small number of complications were identified and all recorded events were mechanical in nature, complication data were treated as a secondary descriptive outcome rather than as a primary modeled endpoint. Radiographic outcomes included postoperative Cobb angle, absolute coronal correction, correction percentage, and, in the subset with paired sagittal measurements, change in kyphosis angle.

### 2.6. Statistical Analysis

Statistical analysis was performed after data cleaning and group verification. Age at first surgery was calculated as the difference between the year of first surgery and the year of birth. Follow-up duration was defined as the interval between the date of first surgery and the date of the last documented clinical and/or radiographic follow-up. Surgical burden per patient-year was calculated as the total number of surgical procedures divided by follow-up duration in years and was assessed in patients with available postoperative follow-up. High surgical burden was defined as ≥3 operations.

Continuous variables are presented as mean ± standard deviation, and categorical variables as counts and percentages. Between-group comparisons were performed using the Mann–Whitney U test for continuous variables and the chi-square test or Fisher’s exact test for categorical variables, as appropriate. Pre- and postoperative radiographic parameters were assessed descriptively and, where applicable, using the paired *t*-test or Wilcoxon signed-rank test, as appropriate.

To account for unequal follow-up duration, the number of operations was additionally analyzed using Poisson regression with log(follow-up) as an offset term. The association between age at first surgery and high surgical burden (≥3 operations) was assessed using logistic regression. Linear regression was used to evaluate predictors of deformity correction percentage. Results are reported as incidence rate ratios (IRRs), odds ratios (ORs), regression coefficients (β), 95% confidence intervals (95% CIs), and *p*-values. A two-sided *p*-value < 0.05 was considered statistically significant.

### 2.7. Ethics

The study was conducted in accordance with the Declaration of Helsinki, and approved by the Local Ethics Committee of the National Scientific Center of Traumatology and Orthopedics named after Academician N.D. Batpenov (Protocol No. 4/1 of 8 November 2023). 

## 3. Results

### 3.1. Cohort Characteristics

After data cleaning and verification according to the predefined analytical criteria, 13 patients were included in the ≤6 years group and 19 patients in the 7–12 years group. The mean age at first surgery was significantly lower in the younger group (3.8 ± 1.5 vs. 9.9 ± 1.6 years, *p* < 0.001), whereas follow-up duration did not differ significantly between groups (2.9 ± 2.1 vs. 2.4 ± 1.4 years, *p* = 0.43).

Baseline coronal deformity magnitude was similar between groups. The mean preoperative Cobb angle was 45.2 ± 19.0° in children aged ≤6 years and 43.1 ± 21.6° in children aged 7–12 years (*p* = 0.61). Kyphoscoliosis was present in 7/13 (53.8%) children in the younger group and 11/19 (57.9%) in the older group. Associated anomalies were recorded in 9/13 (69.2%) patients in the younger group and 7/19 (36.8%) in the older group (*p* = 0.14). The distribution of recorded extra- and intraspinal anomalies by age group is presented in Table 1. Because several anomaly categories contained small numbers, these data were interpreted descriptively and were not included in the primary multivariable models.

### 3.2. Treatment Burden

The total number of operations was numerically higher in the younger group, but the difference was not statistically significant (2.15 ± 1.5 vs. 1.63 ± 0.68, *p* = 0.21). After adjustment for follow-up duration, the number of operations per patient-year was also similar between groups (0.68 ± 0.45 vs. 0.59 ± 0.28, *p* = 0.58). However, the proportion of patients with high surgical burden (≥3 operations) was significantly greater in the ≤6 years group than in the 7–12 years group (38.5% vs. 10.5%, *p* = 0.048). Very high burden (≥4 surgeries) was observed only in the younger group (3/13, 23.1% vs. 0/19, *p* = 0.053). The mean number of hospitalizations showed a similar pattern (3.5 ± 2.3 vs. 2.5 ± 1.4, *p* = 0.15).

### 3.3. Complications

A total of 2 of 32 patients (6.2%) experienced at least one recorded complication during follow-up, and all documented complications were mechanical in nature. Mechanical complications were observed in 1/13 (7.7%) children in the ≤6 years group and in 1/19 (5.3%) children in the 7–12 years group; this between-group difference was not statistically significant (*p* = 1.00). In total, 3 mechanical complication episodes were recorded (2 in the younger group and 1 in the older group). All events were related to the mechanical instability of the construct, including loosening and/or implant breakage. The reoperations performed for these events did not require a change in the overall treatment strategy and were not associated with a meaningful loss of correction.

### 3.4. Radiographic Outcomes

**Figure 1:**
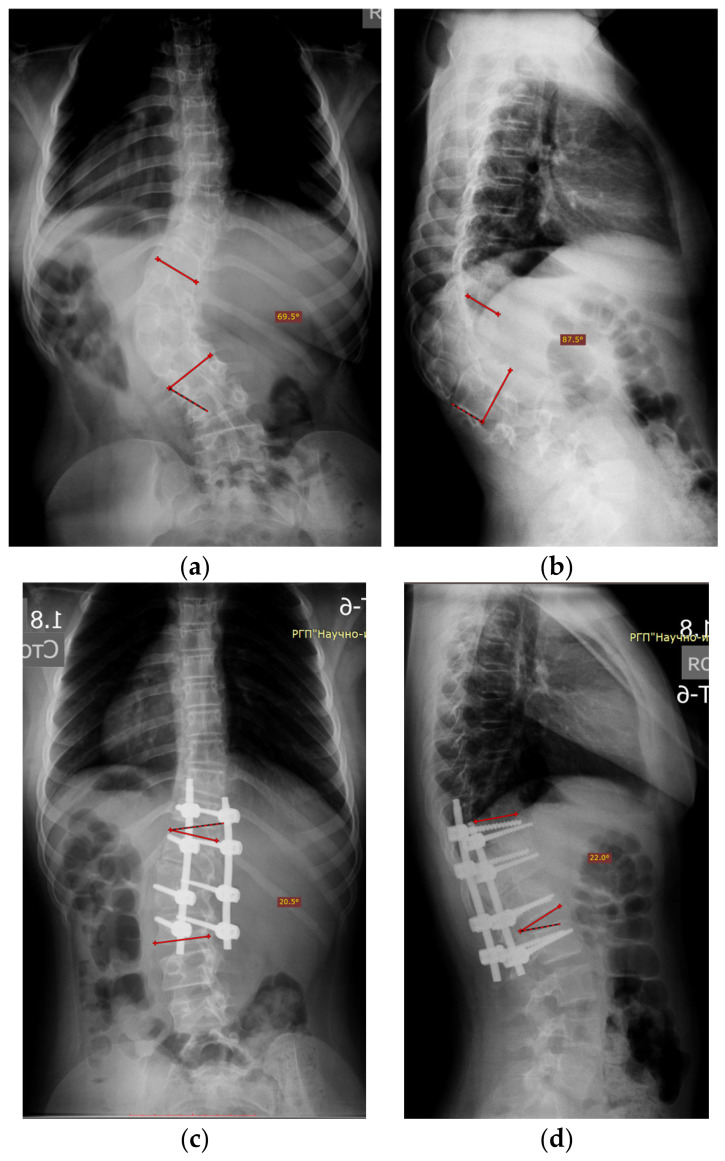
Representative case of congenital spinal deformity correction: (**a**) Preoperative anteroposterior radiograph demonstrating congenital spinal deformity associated with a structural vertebral anomaly; (**b**) preoperative lateral radiograph; (**c**) postoperative anteroposterior radiograph after posterior instrumented correction and fusion showing improved coronal alignment; (**d**) postoperative lateral radiograph showing improved sagittal alignment after correction.

Both age groups showed significant within-group improvement in coronal deformity after surgery. In children aged ≤6 years, the mean Cobb angle improved from 45.2 ± 19.0° preoperatively to 18.9 ± 17.5° at the standardized early postoperative assessment (within-group *p* < 0.001), corresponding to a mean correction percentage of 61.5 ± 35.2%. In children aged 7–12 years, the mean Cobb angle improved from 43.1 ± 21.6° to 14.7 ± 11.3° (within-group *p* < 0.001), with a mean correction percentage of 64.8 ± 30.6%. No statistically significant between-group differences were found in postoperative Cobb angle (*p* = 0.42) or correction percentage (*p* = 0.78). Thus, early coronal radiographic correction appeared broadly comparable between the two age groups. A representative radiographic example of congenital spinal deformity correction from the included cohort is shown in Figure 1.

### 3.5. Kyphosis Subset Analysis

Paired preoperative and postoperative kyphosis measurements were available in 18 patients (7 in the younger group and 11 in the older group). In the ≤6 years group, mean kyphosis improved from 58.4° to 25.6° (*p* = 0.008). In the 7–12 years group, mean kyphosis improved from 49.5° to 23.0° (*p* = 0.031). The magnitude of kyphosis correction did not differ significantly between age groups (*p* = 0.48). These findings support significant within-group sagittal improvement but do not demonstrate an age-related difference in sagittal correction.

### 3.6. Multivariable Regression Analysis

Multivariable regression models were constructed to assess the independent association of age at first surgery with treatment burden and radiographic correction, while adjusting for baseline Cobb angle and kyphosis status (Table 2). Because total follow-up varied between patients, the number of operations was analyzed using Poisson regression with log(follow-up) as an offset term. In this model, age at first surgery was not significantly associated with the rate of operations over time (IRR = 0.96, 95% CI: 0.89–1.03, *p* = 0.27).

In logistic regression for high surgical burden (≥3 operations), older age at first surgery was associated with a lower likelihood of high burden (OR = 0.78 per additional year, 95% CI: 0.61–0.99, *p* = 0.042). When analyzed categorically, patients operated at ≤6 years had more than fivefold higher odds of undergoing ≥3 operations compared with those operated at 7–12 years (OR = 5.14, 95% CI: 1.02–25.9, *p* = 0.047). Baseline Cobb angle and kyphosis status were not independent predictors of high surgical burden.

Linear regression showed that neither age at first surgery (β = 0.92, 95% CI: −2.1 to 3.9, *p* = 0.54) nor baseline Cobb angle (β = −0.09, 95% CI: −0.68 to 0.50, *p* = 0.76) was associated with the percentage of deformity correction.

## 4. Discussion

The present study evaluated whether age at initial surgery is associated with cumulative treatment burden and radiographic outcomes in children with congenital spinal deformities associated with multiple vertebral anomalies. Three main findings emerged. First, both age groups achieved significant coronal correction after surgery. Second, children who underwent initial surgery at ≤6 years experienced a greater cumulative treatment burden, most clearly reflected in the higher frequency of high surgical burden. Third, in adjusted analyses, age at initial surgery appeared to be more closely related to treatment burden than to the magnitude of early coronal correction. Taken together, these findings suggest that, in this patient population, the timing of surgery may be more relevant to longitudinal treatment burden than to early postoperative radiographic correction.

The most clinically relevant observation of this study is the association between younger age at initial surgery and greater cumulative burden of care. In descriptive analyses, children treated at ≤6 years had higher mean numbers of surgeries and hospitalizations, and high surgical burden was more frequent in this group. In adjusted analysis, older age at first surgery was associated with lower odds of high surgical burden. Although this association reached statistical significance in the present sample, it should be interpreted cautiously. The number of high-burden events was limited, and the confidence intervals were wide, particularly in the logistic model, indicating limited estimate precision and possible model instability. Accordingly, the multivariable logistic analysis is best regarded as exploratory and supportive rather than definitive. Nevertheless, the overall direction of the findings was consistent across descriptive and adjusted analyses and supports the interpretation that earlier entry into surgical treatment may be associated with a more intensive longitudinal treatment course.

This pattern remains biologically and clinically plausible. Younger children have greater residual spinal growth, a longer remaining treatment horizon, and more time during which implant-related complications, progression-related events, or additional interventions may occur. In the broader early-onset scoliosis literature, growth-preserving strategies are well known to be associated with repeated procedures, higher complication rates, and substantial family burden [3,7,8,14]. However, our cohort differs from these studies because planned staged procedures and growth-friendly techniques were excluded, and all patients underwent conventional posterior corrective surgery with instrumented fusion. Therefore, the present findings should not be interpreted as evidence against early surgery when clinically indicated. Rather, they suggest that even after successful early radiographic correction, younger children may require a more intensive long-term treatment pathway, including additional procedures, repeated hospital encounters, and prolonged surveillance during growth. This point is clinically relevant for individualized surgical decision-making and preoperative family counseling.

The radiographic findings should be interpreted separately from the burden outcomes. Both age groups showed marked within-group improvement in Cobb angle, but no significant between-group differences were identified in postoperative Cobb angle or correction percentage at the standardized early postoperative assessment. In addition, no baseline variable independently predicted correction percentage in the adjusted linear regression model. These findings suggest that, within the limits of this cohort, age at initial surgery did not substantially influence the ability to achieve meaningful early coronal correction. Clinically, this distinction is important because it implies that the consequences of earlier versus later surgery in this setting may be expressed more through the longitudinal treatment trajectory than through the degree of early radiographic correction.

Our radiographic findings differ from reports in more morphologically uniform populations, particularly isolated hemivertebra cohorts, in which earlier surgery has been associated with greater deformity correction and more favorable long-term radiographic results [10,16]. This discrepancy is not unexpected. The present cohort was intentionally broader and included children with multiple vertebral anomalies rather than isolated single-level deformities. In addition, the current study excluded staged growth-preserving constructs and focused on conventional posterior corrective surgery, thereby reducing treatment heterogeneity while also making the cohort fundamentally different from highly selected hemivertebra-resection series. Therefore, the absence of an age-related difference in correction in our data should not be interpreted as contradicting those studies, but rather as suggesting that the influence of age may depend strongly on deformity morphology, treatment strategy, and the outcome domain being examined.

An additional observation of the present study is that the recorded complication profile was narrow: all documented complications were mechanical, and no non-mechanical complications were identified in the dataset. Mechanical complications were numerically more frequent in children who underwent initial surgery at ≤6 years; however, the absolute number of affected patients was very small, and the between-group difference was not statistically significant. These findings should therefore be interpreted descriptively rather than as evidence of an independent age-related effect on complication risk. At the same time, the fact that all recorded complications were mechanical supports the interpretation that, in this cohort, the additional treatment burden was driven primarily by implant- or construct-related events requiring further management rather than by infectious, neurologic, or systemic postoperative complications. Importantly, the reoperations performed for these events did not require a change in the overall treatment strategy and were not associated with a meaningful loss of correction. This distinction is clinically relevant because it suggests that the observed burden signal in younger children may relate more to mechanical durability and the longer treatment trajectory than to a broader difference in perioperative safety profile [7].

Another relevant observation is the absence of stable associations between cumulative burden and the exploratory morphological variables assessed in this study, including localization, deformity type, kyphosis status, and associated anomalies. This should not be taken as evidence that these factors are clinically unimportant. Congenital spinal deformity is intrinsically heterogeneous, and both deformity morphology and associated anomalies are known to influence evaluation, risk stratification, and treatment planning [1,6]. In the present dataset, the more likely explanation is limited statistical power together with sparse subgroup counts, particularly for localization categories. The same consideration applies more broadly to the negative findings in the multivariable models.

The sagittal-plane findings also require cautious interpretation. In the paired subset with available kyphosis measurements, both age groups showed significant within-group improvement, indicating that surgery was effective not only for coronal correction but also for sagittal deformity control when kyphosis was present. However, no statistically significant between-group difference in kyphosis correction was detected. Given the limited number of patients with paired sagittal data, the most appropriate interpretation is that both groups improved sagittally, but the present study does not provide evidence for an age-related difference in sagittal correction.

Respiratory function is another clinically important outcome domain in pediatric spinal deformity surgery. This issue is particularly relevant in congenital spinal deformities because, unlike adolescent idiopathic scoliosis, congenital deformities may be associated with vertebral malformations, thoracic cage abnormalities, rib anomalies, and deformities located in the thoracic spine or transitional junctions. Therefore, findings from AIS cohorts cannot be directly extrapolated to children with congenital scoliosis or kyphoscoliosis. Although posterior correction in mild to moderate AIS may not necessarily result in significant improvement in pulmonary function during follow-up [17], the relationship between deformity correction and respiratory development in congenital deformities may be different and requires separate evaluation. In the present study, pulmonary function was not systematically assessed; therefore, we could not determine whether radiographic correction translated into respiratory benefit or whether respiratory outcomes differed between younger and older patients.

From a practical perspective, the present findings support a broader framework for preoperative counseling in congenital spinal deformity. In routine practice, discussions with families often focus on curve severity, technical aspects of surgery, and expected radiographic correction [8]. The current data suggest that the cumulative burden of care should also be addressed explicitly. In younger children, even when satisfactory deformity correction is achieved, the overall treatment pathway may still involve more hospitalizations and a greater likelihood of additional procedures. This perspective is consistent with the broader early-onset scoliosis literature, which increasingly recognizes repeated healthcare encounters and indirect family burden as important outcome domains [3,7,8].

An important methodological consideration in the present study is the heterogeneity of follow-up duration across patients. Because children operated at a younger age may remain under observation for a longer period, unequal follow-up has the potential to influence the cumulative number of procedures recorded over time. To address this issue, follow-up duration was defined as the interval from the first surgery to the last documented clinical and/or radiographic follow-up, and the number of operations was additionally analyzed using a Poisson model with log(follow-up) as an offset term. This approach improves comparability of operation rates between patients with different lengths of observation; however, it does not fully eliminate the limitations inherent to retrospective follow-up. In particular, differences in surveillance intensity, timing of visits, and the relatively small sample size may still affect burden estimates. Accordingly, the findings related to cumulative surgical burden should be interpreted cautiously and confirmed in larger cohorts with standardized follow-up schedules.

This study has several limitations. First, it is a retrospective single-center study with a relatively small sample size, which limits generalizability and reduces the stability of subgroup and multivariable analyses. Second, although operation counts were adjusted for individual follow-up duration defined from the first surgery to the last documented clinical and/or radiographic follow-up, follow-up was not fully uniform across patients, and retrospective ascertainment may have introduced variability in the intensity and timing of postoperative surveillance. Third, several clinically relevant categories, particularly deformity localization and specific associated anomaly subtypes, contained sparse counts and therefore could not be robustly modeled. Fourth, kyphosis analyses were restricted to a reduced paired subset, and the study did not include standardized patient-reported outcomes, formal assessments of caregiver burden, or systematic pulmonary function testing. Therefore, we could not determine whether radiographic correction translated into respiratory benefit or whether respiratory outcomes differed according to age at initial surgery. Fifth, hidden blood loss and the ratio between hidden and total blood loss were not assessed, although hidden blood loss may represent a clinically relevant component of total bleeding in congenital scoliosis surgery [18]. Because of the retrospective design, the available dataset did not contain sufficiently standardized perioperative laboratory, transfusion, and intraoperative blood loss data to calculate hidden blood loss reliably; therefore, we could not determine whether younger age at surgery was associated with differences in hidden blood loss. Finally, although the overall surgical strategy was more homogeneous than in many early-onset scoliosis series, some variability in implant configuration and surgeon-level decision-making remained. These limitations should be considered when interpreting both the observed association between younger age at initial surgery and greater treatment burden and the absence of significant predictors of radiographic correction.

Despite these limitations, the study has important strengths. It addresses a clinically relevant but comparatively underexplored question: whether the timing of surgery in congenital deformity influences not only radiographic correction but also the cumulative burden of care. It also examines a real-world cohort of children with multiple vertebral anomalies rather than a narrowly selected single-morphology group and applies adjusted regression modeling in addition to simple group comparisons. In this respect, the study helps refine the distinction between radiographic success and longitudinal treatment burden, an outcome domain of increasing relevance in pediatric spinal deformity care.

## 5. Conclusions

In this cohort of children with congenital spinal deformities associated with multiple vertebral anomalies, conventional posterior instrumented fusion achieved substantial early radiographic improvement in both age groups. Although coronal correction was significant and broadly comparable, younger age at initial surgery was associated with a higher likelihood of high surgical burden, whereas the time-adjusted rate of operations and early coronal correction did not differ significantly between groups. These findings suggest that, in congenital spinal deformity, the timing of surgery may influence longitudinal treatment burden more than the magnitude of early radiographic correction.

These results should nevertheless be interpreted cautiously in view of the retrospective single-center design, limited sample size, small number of high-burden events, and variability in follow-up. Larger multicenter studies with standardized follow-up protocols are needed to confirm these findings and to further refine age-informed decision-making in the surgical management of congenital spinal deformity.

## Figures and Tables

**Table 1:** Associated extra- and intraspinal anomalies by age group.

Associated Anomaly	≤6 Years Group, n/N (%)	7–12 Years Group, n/N (%)	Total, n/N (%)
Any associated anomaly	9/13 (69.2%)	7/19 (36.8%)	16/32 (50.0%)
Cardiovascular/situs anomalies	7/13 (53.8%)	4/19 (21.1%)	11/32 (34.4%)
Intraspinal anomalies/spinal dysraphism	2/13 (15.4%)	2/19 (10.5%)	4/32 (12.5%)
Rib/thoracic cage anomalies	1/13 (7.7%)	1/19 (5.3%)	2/32 (6.3%)
Gastrointestinal anomalies	1/13 (7.7%)	0/19 (0.0%)	1/32 (3.1%)
Craniofacial/ear anomalies	1/13 (7.7%)	1/19 (5.3%)	2/32 (6.3%)
Limb anomalies	1/13 (7.7%)	0/19 (0.0%)	1/32 (3.1%)
Other associated congenital or syndromic features	0/13 (0.0%)	3/19 (15.8%)	3/32 (9.4%)

Note: Percentages were calculated within each age group. Some patients had more than one associated anomaly; therefore, category totals may exceed the number of patients with any associated anomaly. Cardiovascular/situs anomalies included minor cardiac variants, congenital heart disease, dextrocardia, and situs-related abnormalities. Intraspinal anomalies/spinal dysraphism included diastematomyelia, spina bifida, syringomyelia, and other recorded dysraphism-related findings. Other associated congenital or syndromic features included Klippel–Feil syndrome and joint hypermobility syndrome.

**Table 2:** Multivariable regression models for surgical burden and radiographic correction.

Predictor	Poisson Model for Rate of Surgeries, IRR (95% CI)	*p*-Value	Logistic Model for ≥3 Surgeries, OR (95% CI)	*p*-Value	Linear Model for Correction %, β (95% CI)	*p*-Value
Age at first surgery, per year	0.96 (0.89–1.03)	0.27	0.78 (0.61–0.99)	0.042	0.92 (−2.1 to 3.9)	0.54
Baseline Cobb angle, per degree	1.004 (0.994–1.014)	0.45	1.02 (0.97–1.07)	0.38	−0.09 (−0.68 to 0.50)	0.76
Kyphosis present	1.10 (0.68–1.78)	0.69	1.05 (0.20–5.49)	0.95	2.80 (−18.5 to 24.1)	0.79

Abbreviations: IRR, incidence rate ratio; OR, odds ratio; CI, confidence interval. The Poisson model included log(follow-up) as an offset term.

## Data Availability

The data presented in this study are available on request from the corresponding author due to restrictions related to patient confidentiality and institutional ethical requirements.

## Abbreviations

The following abbreviations are used in this manuscript:

IRR	incidence rate ratio
OR	odds ratio
CI	confidence interval

## References

[1] Sebaaly A., Daher M., Salameh B., Ghoul A., George S., Roukoz S. (2022). Congenital scoliosis: A narrative review and proposal of a treatment algorithm. EFORT Open Rev..

[2] Wu N., Liu L., Zhang Y., Wang L., Wang S., Zhao S., Li G., Yang Y., Lin G., Shen J. (2023). Retrospective analysis of associated anomalies in 636 patients with operatively treated congenital scoliosis. J. Bone Jt. Surg. Am..

[3] Johnson A.N., Lark R.K. (2024). Current concepts in the treatment of early-onset scoliosis. J. Clin. Med..

[4] Abdaliyev S., Yestay D., Baitov D. (2024). Correction of a congenital kyphoscoliosis associated with diastematomyelia. J. Surg. Case Rep..

[5] Yin X.J., Li Z.Q., Li G.Z., Chen G.L., Xu K.X., Zhu Y.P., Zhang J.G., Wu N. (2024). The multisystem deformities features of Klippel-Feil syndrome patients combined with congenital scoliosis. Zhonghua Yi Xue Za Zhi.

[6] Latalski M., Fatyga M., Sowa I., Wojciak M., Starobrat G., Danielewicz A. (2021). Complications in growth-friendly spinal surgeries for early-onset scoliosis: Literature review. World J. Orthop..

[7] Li Y., Swallow J., Gagnier J., Cahill P.J., Sponseller P.D., Garg S., Thompson G.H., Ramo B.A. (2021). Pediatric Spine Study Group. Growth-friendly surgery results in more growth but a higher complication rate and unplanned returns to the operating room compared to single fusion in neuromuscular early-onset scoliosis: A multicenter retrospective cohort study. Spine Deform..

[8] McFadden R.J., Hauth L., Gregoski M., Anari J.B., Brooks J.T., Sawyer J.R., Marshall M., Murphy R.F., Pediatric Spine Study Group (2024). A multicenter evaluation of the time and travel burden on families with children treated for early-onset scoliosis. Spine Deform..

[9] Campbell M., Matsumoto H., St Hilaire T., Roye B.D., Roye D.P., Vitale M.G. (2020). Burden of care in families of patients with early-onset scoliosis. J. Pediatr. Orthop. B.

[10] Chang D.G., Suk S.I., Kim J.H., Ha K.Y., Na K.H., Lee J.H. (2015). Surgical outcomes by age at the time of surgery in the treatment of congenital scoliosis in children under age 10 years. Spine J..

[11] Tsirikos A.I., Lipton G., Chang W.N., Dabney K.W., Miller F. (2008). Surgical correction of scoliosis in pediatric patients with cerebral palsy using the unit rod instrumentation. Spine (Phila. Pa 1976).

[12] Zhang Y., Wang Y., Xie J., Bi N., Zhao Z., Li T., Shi Z., Huang T., Gao B., Gu K. (2022). Factors associated with postoperative respiratory complications following posterior spinal instrumentation in children with early-onset scoliosis. Orthop. Surg..

[13] Furdock R., Brouillet K., Luhmann S.J. (2019). Organ system anomalies associated with congenital scoliosis: A retrospective study of 305 patients. J. Pediatr. Orthop..

[14] Li Y., Liu W., Shi B., Liu Z., Mao S., Qiao J., Zhu Z., Qiu Y. (2025). Repeated vertebral column resection (Re-VCR) in congenital scoliosis with curve progression after instrumentation removal. Orthop. Surg..

[15] Zhang H.Q., Xiao L.G., Guo C.F., Wang Y.X., Wu J.H., Liu J.Y. (2021). Deformed complex vertebral osteotomy technique for management of severe congenital spinal angular kyphotic deformity. Orthop. Surg..

[16] Guo S., Zheng Y., Zhang Z., Fu D., Wang J., Li H., Qian C., Wang D. (2024). Evaluation of the efficacy of posterior hemivertebrectomy combined with two or more segments fusion. BMC Musculoskelet. Disord..

[17] Kato S., Murray J.C., Ganau M., Tan Y., Oshima Y., Tanaka S. (2019). Does Posterior Scoliosis Correction Improve Respiratory Function in Adolescent Idiopathic Scoliosis? A Systematic Review and Meta-analysis. Glob. Spine J..

[18] Raitio A., Heiskanen S., Soini V., Helenius L., Syvänen J., Helenius I. (2024). Hidden blood loss and bleeding characteristics in children with congenital scoliosis undergoing spinal osteotomies. Int. Orthop..

